# Quality, content structure, and reliability of prostatitis-related information on short-video platforms: a cross-platform analysis of TikTok and Bilibili

**DOI:** 10.1186/s12894-026-02225-y

**Published:** 2026-06-17

**Authors:** Pan Ding, Qinghua Luo, Leihua Cao

**Affiliations:** Department of Urology, Nanchang People’s Hospital, Nanchang City, 330000 People’s Republic of China

**Keywords:** Prostatitis, TikTok, Bilibili, Digital health, Health information quality, Short video platforms

## Abstract

**Background:**

Prostatitis, particularly chronic prostatitis/chronic pelvic pain syndrome (CP/CPPS), remains a common yet challenging condition in urological practice, largely due to its heterogeneous etiology, diagnostic complexity, and high levels of patient anxiety. Increasingly, patients seek information about prostatitis from short-video platforms; however, the quality and clinical relevance of such content remain unclear.

**Methods:**

A cross-sectional content analysis was conducted to evaluate prostatitis-related videos on TikTok and Bilibili. Video characteristics, engagement metrics, uploader type, and content themes were extracted. Information quality and reliability were assessed using the Global Quality Scale (GQS) and the modified DISCERN (mDISCERN) instrument, while content completeness was evaluated using a six-domain clinical information framework. Comparisons were performed across platforms and uploader categories, and correlations between engagement metrics and quality indicators were analyzed.

**Results:**

A total of 223 videos were included. Overall information quality and reliability were moderate. Content distribution was heavily skewed toward treatment-related information, whereas disease awareness, diagnostic principles, and preventive or long-term management content were markedly underrepresented. Videos uploaded by healthcare professionals demonstrated significantly higher quality, reliability, and content completeness than those from non-professional sources. TikTok videos achieved higher overall quality and reliability scores compared with Bilibili. Engagement metrics showed only weak correlations with information quality and reliability indicators.

**Conclusion:**

Prostatitis-related information on short-video platforms is characterized by moderate quality and substantial structural imbalance, with an overemphasis on treatment and insufficient coverage of diagnostic reasoning and long-term management. From a urological clinical perspective, incomplete or misleading online information may shape patient expectations and has the potential to complicate clinical communication and shared decision-making, particularly regarding diagnostic evaluation, antibiotic expectations, and long-term management. Greater involvement of urologists in structured digital patient education and the promotion of clinically accurate content are needed to mitigate misinformation and improve patient understanding.

**Supplementary Information:**

The online version contains supplementary material available at 10.1186/s12894-026-02225-y.

## Introduction

Prostatitis is one of the most prevalent urological disorders in men. Epidemiological studies have reported a lifetime prevalence ranging from approximately 2% to 10%, reaching up to 15% in certain populations, and it accounts for a substantial proportion of urology outpatient visits [1–4]. Among all cases, chronic prostatitis/chronic pelvic pain syndrome (CP/CPPS) constitutes more than 90%, characterized by complex etiologies, heterogeneous clinical manifestations, and a high recurrence rate [4–7]. The pathogenesis of CP/CPPS involves multiple interacting mechanisms, including infection, immune-inflammatory responses, dysregulation of neural pathways, and pelvic floor dysfunction, which collectively complicate clinical classification, differential diagnosis, and comprehensive management strategies. These challenges substantially impair patients’ quality of life and impose significant psychological and economic burdens [8, 9]. Against this background, patient demand for reliable information regarding disease understanding, diagnostic pathways, therapeutic options, and long-term management has continuously increased. Concurrently, information acquisition is progressively shifting from traditional healthcare settings toward digital platforms, with digital media becoming deeply embedded in the formation of patients’ disease cognition frameworks and health-related decision-making processes.

Consequently, short-video platforms have become core infrastructures within the digital health ecosystem, serving as primary gateways for public access to health-related information [10]. Platforms such as TikTok and Bilibili, supported by algorithmic curation systems, high user engagement, and large-scale audience penetration, are playing an increasingly central role in contemporary health communication. Nevertheless, existing studies consistently indicate that health content on these platforms is often characterized by fragmented narratives, limited evidence-based support, and weak medical structuring. Furthermore, content visibility and dissemination intensity frequently fail to reflect scientific rigor, which may systematically enhance the exposure and influence of inaccurate or low-credibility information [11–13].

This structural distortion is particularly relevant in the context of chronic and symptom-based diseases, where incomplete or poorly structured information may shape patients’ disease perception, treatment expectations, and health-seeking decisions before clinical consultation [14]. Prostatitis, particularly chronic prostatitis/chronic pelvic pain syndrome (CP/CPPS), represents a clinically important example. Unlike many acute or clearly defined urological conditions, CP/CPPS is characterized by heterogeneous symptoms, diagnostic uncertainty, multifactorial pathophysiology, recurrent symptoms, and the absence of a single definitive curative treatment. These features make patients particularly vulnerable to fragmented online information, especially content that overemphasizes rapid symptom relief, medication-based treatment, or simplified causal explanations while providing insufficient discussion of diagnostic reasoning, differential diagnosis, psychological factors, pelvic floor dysfunction, and long-term multimodal management.

Although previous studies have evaluated the quality of short-video content across various disease contexts, systematic investigations focusing on prostatitis remain limited. More importantly, existing social media health information studies have often emphasized overall quality or reliability scores, while paying less attention to whether the content structure aligns with disease-specific clinical needs. For prostatitis, the clinical value of online information depends not only on general quality and reliability, but also on whether videos provide balanced coverage of disease cognition, diagnostic principles, treatment indications, and long-term management. Therefore, a disease-specific, clinically oriented assessment of prostatitis-related short videos is warranted.

Accordingly, this study conducted a systematic content analysis of prostatitis-related short videos on two major Chinese short-video platforms, TikTok and Bilibili, to characterize dissemination patterns, uploader characteristics, thematic distributions, information quality, reliability, and disease-specific content completeness. By integrating GQS, mDISCERN, and a six-domain clinical content framework, this study aimed to move beyond a general descriptive assessment of video quality and to identify clinically meaningful structural gaps in prostatitis-related digital health communication. The findings may provide practical implications for urologists, healthcare institutions, and platform governance by informing the development of more structured, evidence-based, and patient-centered digital education strategies for prostatitis.

## Methods

### Study design

This study adopted a cross-sectional infodemiology-based content analysis design to evaluate the quality, reliability, and medical content coverage of prostatitis-related health information disseminated on short-video platforms.

### Data sources and search strategy

On January 2, 2026, a systematic cross-sectional search was conducted on the Chinese domestic version of TikTok and Bilibili using the search term“prostatitis”. Both platforms were accessed in the Chinese domestic short-video environment. The same keyword was applied to both platforms to maintain consistency in the cross-platform search strategy. The general term “prostatitis” was selected as the primary keyword because it broadly captures prostatitis-related information that users may encounter during routine platform searches. Although chronic prostatitis/chronic pelvic pain syndrome (CP/CPPS) is the most common and clinically relevant subtype, terms such as “chronic prostatitis” and “CP/CPPS” were not used as additional independent search terms because the aim of this study was to evaluate the general prostatitis-related information environment rather than a narrower subtype-specific information subset. Searches were performed on the same day using desktop computers to obtain a standardized cross-sectional snapshot and to reduce temporal variation in platform algorithms and content popularity. Hashtags were not used as independent search terms; however, videos retrieved through the platform search results were eligible for inclusion regardless of whether hashtags appeared in their titles, captions, or descriptions. To minimize the influence of personalized recommendation algorithms, newly registered accounts were used on both platforms, and browsing histories, cache data, and prior interaction records were cleared before retrieval. No manual sorting or filtering options were applied. Videos were ranked according to each platform’s default comprehensive sorting algorithm, which reflects platform-specific relevance and recommendation logic rather than manual sorting by a single metric such as views, likes, comments, or shares. The top 150 videos from each platform were collected as the initial sample, resulting in 300 videos for initial screening.

### Inclusion and exclusion criteria

Inclusion criteria were as follows: (1) content directly related to prostatitis; (2) videos presented in Chinese or English; (3) independently playable short-video format; and (4) audiovisual quality sufficient for content evaluation.

Exclusion criteria included: (1) content unrelated to prostatitis; (2) duplicate uploads; (3) explicit commercial or promotional content; and (4) videos with severely compromised audiovisual quality that precluded reliable assessment.

After screening, a total of 223 videos were included in the final analysis: 115 videos from TikTok (excluding 26 unrelated videos and 9 advertisements) and 108 videos from Bilibili (excluding 17 advertisements, 11 unrelated videos, and 14 duplicate videos).

### Data extraction

The following variables were extracted for each video: platform source, video duration (seconds), engagement metrics (number of likes, favorites, comments, and shares), uploader type (specialist physicians/healthcare professionals, non-specialist contributors, and individual users), and content dimension labels.

### Uploader classification

Uploaders were classified into three categories according to publicly available account information and video content. Specialists were defined as accounts clearly indicating a professional medical identity related to prostatitis or male genitourinary health, including urologists, andrologists, traditional Chinese medicine andrology practitioners, and videos from verified hospital or medical institution accounts in which the content was presented by urology, andrology, or traditional Chinese medicine andrology physicians. Non-specialists were defined as health-related accounts that provided medical or health education content but did not clearly indicate specialist credentials related to prostatitis, such as general health bloggers, medical science communicators, commercial health-media accounts, or physicians from unrelated specialties. Individual users were defined as personal accounts sharing individual experiences, symptoms, opinions, or non-professional interpretations without identifiable medical credentials.

Uploader identity was determined based on profile descriptions, verification labels, institutional affiliation, stated professional title, username, and consistency between the account identity and video content. Ambiguous accounts were reviewed by two researchers, and disagreements were resolved by a third reviewer. If specialist identity could not be reliably verified, the account was classified conservatively as a non-specialist or individual user.

### Quality and reliability assessment

Overall video quality was evaluated using the Global Quality Scale (GQS) (scores ranging from 1 to 5; Table 1), while information reliability was assessed using the modified DISCERN (mDISCERN) instrument (scores ranging from 0 to 5; Table 2).

**Table 1:** The Global Quality Score (GQS) quality criteria

Item features	Points
Poor quality; poor flow of the videos; most information missing; not at all useful for patients	1
Generally poor quality; some information listed, but many important topics missing; of very limited use to patients	2
Moderate quality; suboptimal flow; some important adequately discussed, but other information poorly discussed; somewhat useful for patients	3
Good quality and generally good flow; most of the relevant information listed, but some topics not covered; useful for patients	4
Excellent quality and flow; very useful for patients	5

**Table 2:** The Modified DISCERN (mDISCERN) quality criteria

Reliability Score	
1. Is the video clear, concise, and understandable?	
2. Are valid sources cited?	
3. Is the content presented balanced and unbiased?	
4. Are additional sources of content listed for patient reference?	
5. Are areas of uncertainty mentioned?	

Zhang et al. [15], Ni et al. [16]. Two trained researchers independently performed the assessments, and any discrepancies were resolved through adjudication by a third reviewer. The GQS and mDISCERN instruments used in this study are previously published assessment tools that have been widely applied in studies evaluating online health information quality. These tools were used for non-commercial academic research purposes with appropriate citation to the original sources. No commercial software, paid scoring system, or restricted-access instrument was used. The six-domain clinical content scoring framework was developed by the authors based on clinically relevant domains of prostatitis-related information and previously published digital health information quality frameworks.

### Video content score

A six-domain medical content scoring framework was developed, encompassing epidemiology, etiology, clinical manifestations, diagnosis, treatment, and prevention. Each domain was scored from 0 to 2 points, yielding a total possible score ranging from 0 to 12 points [17].The scoring procedure followed the same independent evaluation process as the quality and reliability assessments.

### Statistical analysis

Continuous variables were summarized as medians with interquartile ranges (IQRs), and categorical variables were presented as frequencies and proportions. Platform-level comparisons were conducted using the Mann–Whitney U test for continuous variables and the chi-square test or Fisher’s exact test for categorical variables, as appropriate. Differences across uploader categories were assessed using the Kruskal–Wallis test for continuous variables and the chi-square test or Fisher’s exact test for categorical variables, as appropriate. Associations between engagement metrics and quality-related measures were examined using Spearman’s rank correlation coefficients. Inter-rater reliability was assessed across all included videos before final consensus. Intraclass correlation coefficients (ICCs) were calculated for GQS, mDISCERN, and the total video content score using a two-way random-effects model for absolute agreement. Weighted Cohen’s kappa (κ) coefficients were calculated for the six ordinal content-domain scores. All tests were two-sided, with statistical significance set at *P* < 0.05. Exact P values were reported whenever possible. All analyses and visualizations were performed using R software, version 4.3.3.

## Results

### Sample characteristics and overall features

**Fig. 1:**
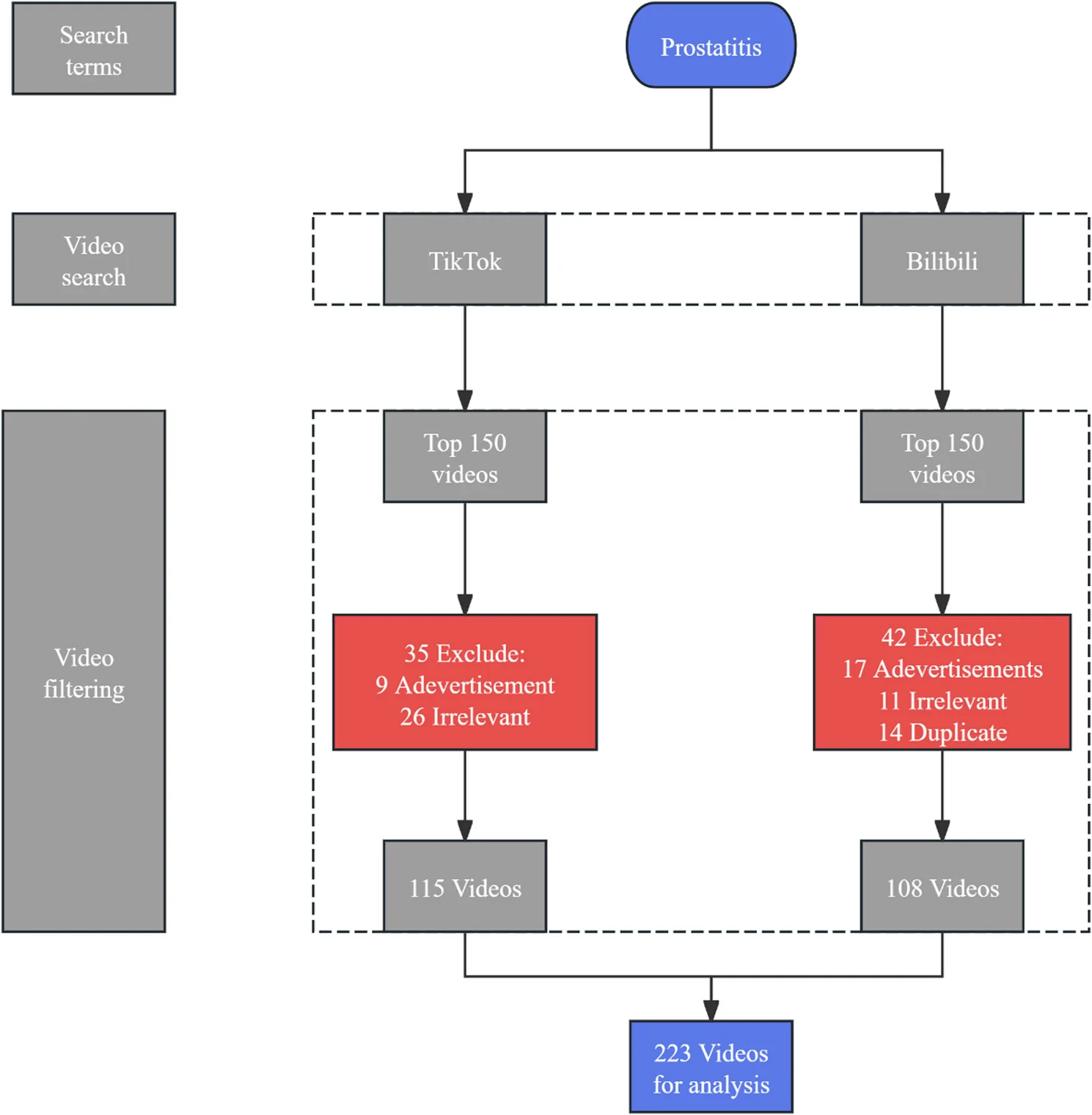
Flowchart of the study. Notation: Videos related to prostatitis were retrieved from TikTok and Bilibili using predefined search terms. A total of 300 videos were initially screened, including the top 150 videos retrieved from TikTok and the top 150 videos retrieved from Bilibili. After exclusion of advertisements, irrelevant content, and duplicates, 115 TikTok videos and 108 Bilibili videos were included, yielding a total of 223 videos for final analysis.

A total of 300 videos were initially screened, including the top 150 videos retrieved from TikTok and the top 150 videos retrieved from Bilibili. After applying the inclusion and exclusion criteria, 223 prostatitis-related short videos were included in the final dataset, comprising 115 videos from TikTok and 108 videos from Bilibili (Fig. 1). Across the overall sample, the median video length was 94.00 s (IQR: 54.00–197.00). Engagement indicators demonstrated median values of 80.00 likes, 27.00 favorites, 9.00 comments, and 9.00 shares. Thematic distribution analysis revealed a pronounced dominance of treatment-oriented content, accounting for 86.55% of all videos.Overall informational quality and credibility were assessed as moderate, with median scores of 3.00 on both the Global Quality Scale (GQS) and the modified DISCERN (mDISCERN) instrument (Table 3).

**Table 3:** General Information,quality,and reliability scores of prostatitis-related videos on TikTok and BiliBili

Variables	Total (*n* = 223)
General information	
Video length(s), M (Q₁, Q₃)	94.00 (54.00, 197.00)
Likes, M (Q₁, Q₃)	80.00 (17.00, 494.50)
Collections, M (Q₁, Q₃)	27.00 (7.00, 236.00)
Comments, M (Q₁, Q₃)	9.00 (2.00, 64.00)
Shares, M (Q₁, Q₃)	9.00 (1.00, 56.00)
Video content *n*(%)	
Epidemiology	56（25.11%）
Etiology	87（39.01%）
Clinical manifestation	103（46.19%）
Diagnosis	80（35.87%）
Treatment	193（86.55%）
Prevention	110（49.33%）
Video quality	
GQS score, M (Q₁, Q₃)	3.00 (2.00, 3.00)
mDISCERN, M (Q₁, Q₃)	3.00 (2.00, 3.00)
Uploader *n*(%)	
Individual user	53 (23.77%)
Non-specialists	36 (16.14%)
Specialists	134 (60.09%)

*GQS* Global Quality Score, *M* Median, *Q₁* 1st Quartile, *Q₃* 3rd Quartile

### Inter-rater reliability

Inter-rater reliability was assessed across all included videos before final adjudication. The ICC values for GQS, mDISCERN, and the total video content score were 0.963, 0.930, and 0.982, respectively, indicating excellent agreement between the two reviewers. The weighted Cohen’s κ values for the six content domains ranged from 0.899 to 0.994, suggesting almost perfect agreement in content-domain classification. Detailed inter-rater reliability results are provided in Supplementary Table S1.

### Platform-based comparisons

**Fig. 2:**
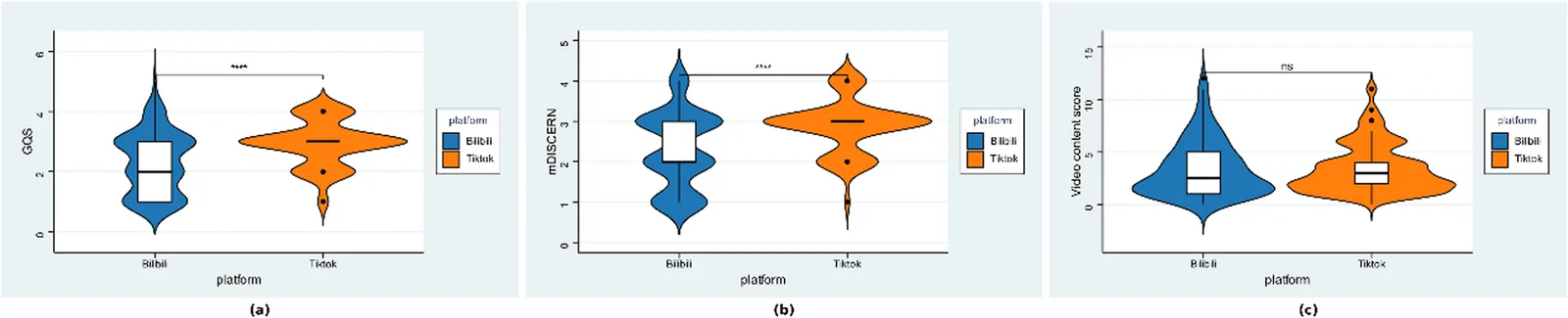
Platform-level differences in GQS, mDISCERN, and video content score. Notation: (**a**) Global Quality Scale (GQS), (**b**) modified DISCERN (mDISCERN), and (**c**) video content scores are compared between Bilibili and TikTok. Data are shown as violin plots with embedded boxplots. Statistical differences are indicated (****P < 0.0001; ns, not significant)

Comparative analyses revealed significant differences between TikTok and Bilibili in both dissemination characteristics and quality-related performance (Table 4, Fig. 2). In terms of engagement, TikTok videos exhibited significantly higher numbers of likes and shares than those on Bilibili, whereas no statistically significant differences were observed for favorites and comments. Regarding video attributes, Bilibili videos were significantly longer in duration compared with TikTok.With respect to information quality and reliability, videos on TikTok achieved significantly higher Global Quality Scale (GQS) scores and modified DISCERN (mDISCERN) scores than those on Bilibili, indicating superior performance in content quality and informational credibility. However, no significant difference was observed between the two platforms in terms of overall medical content coverage scores.

**Table 4:** Comparison of characteristics between different short-video platforms

Variables	Bilibili (*n* = 108)	Tiktok (*n* = 115)	*P*
Video length(s), M (Q₁, Q₃)	175.00 (99.75, 344.25)	65.00 (43.00, 93.00)	**< 0.001**
Likes, M (Q₁, Q₃)	29.00 (5.75, 491.50)	119.00 (39.50, 477.00)	**< 0.001**
Collections, M (Q₁, Q₃)	23.50 (6.00, 352.25)	31.00 (13.00, 186.00)	0.486
Comments, M (Q₁, Q₃)	6.00 (1.00, 148.00)	10.00 (3.00, 30.50)	0.709
Shares, M (Q₁, Q₃)	3.50 (0.00, 90.00)	14.00 (4.00, 50.50)	0.004
mDISCERN, M (Q₁, Q₃)	2.00 (2.00, 3.00)	3.00 (3.00, 3.00)	**< 0.001**
GQS score, M (Q₁, Q₃)	2.00 (1.00, 3.00)	3.00 (3.00, 3.00)	**< 0.001**
Video content(n)%, M (Q₁, Q₃)	2.50 (1.00, 5.00)	3.00 (2.00, 4.00)	0.656
Epidemiology	25(23.15%)	31(26.96%)	-
Etiology	51(47.22%)	36(31.30%)	-
Clinical manifestation	62(57.41%)	41(35.65%)	-
Diagnosis	41(37.96%)	39(33.91%)	-
Treatment	83(76.85%)	110(95.65%)	-
Prevention	51(47.22%)	59(51.30%)	-
Uploader(n)%			**< 0.001**
Individual user	44 (40.74%)	9 (7.83%)	-
Non-specialists	15 (13.89%)	21 (18.26%)	-
Specialists	49 (45.37%)	85 (73.91%)	-

### Uploader structure and source composition

**Fig. 3:**
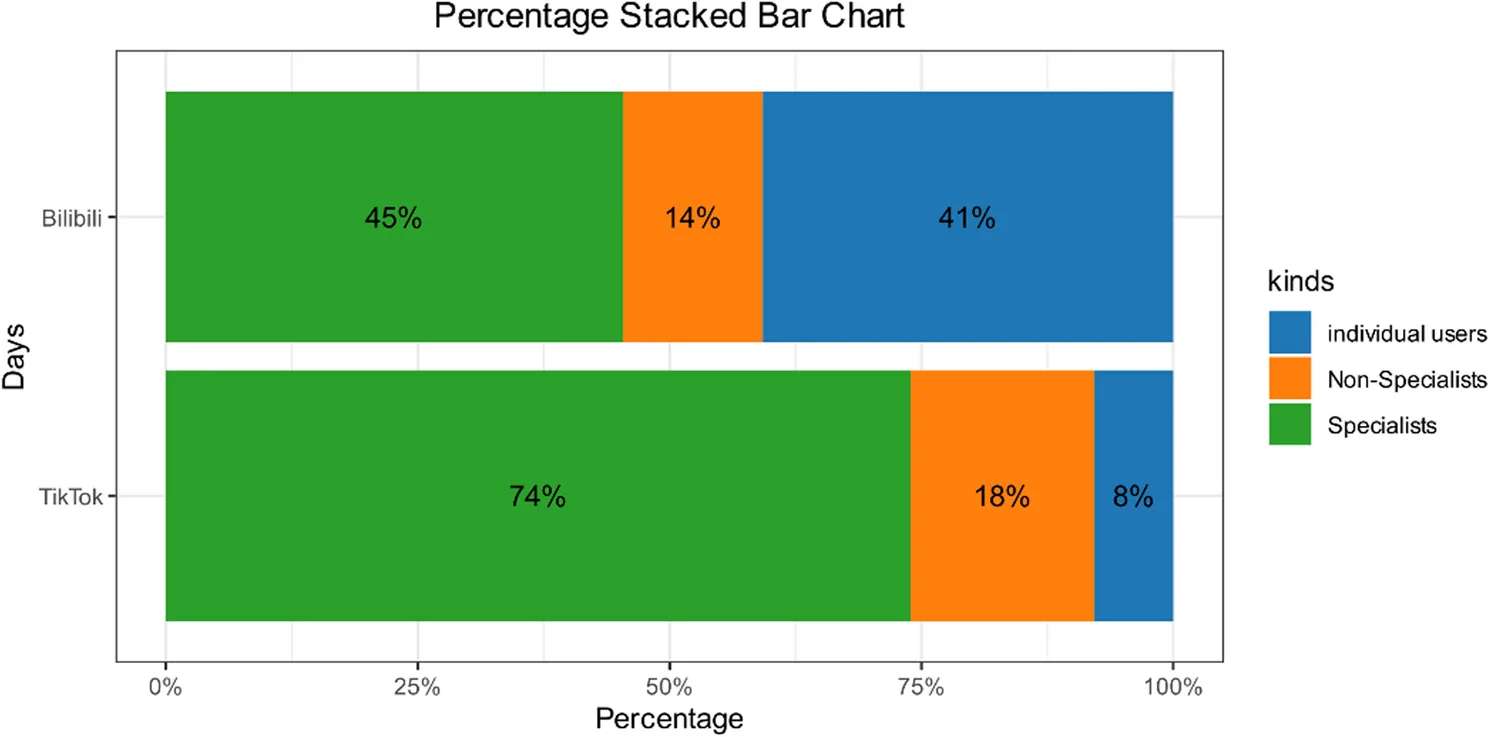
Distribution of videos by uploader type on TikTok and Bilibili platforms

Based on the predefined uploader classification criteria, analysis of uploader composition revealed a pronounced divergence in the sources of health information between the two platforms (Fig. 3). On TikTok, content was predominantly generated by specialist physicians and healthcare professionals, accounting for approximately 74% of all videos. In contrast, Bilibili exhibited a more heterogeneous uploader structure, with comparable proportions of professional accounts and individual users, alongside a relatively higher contribution from non-professional sources.

### Distribution of content themes

**Fig. 4:**
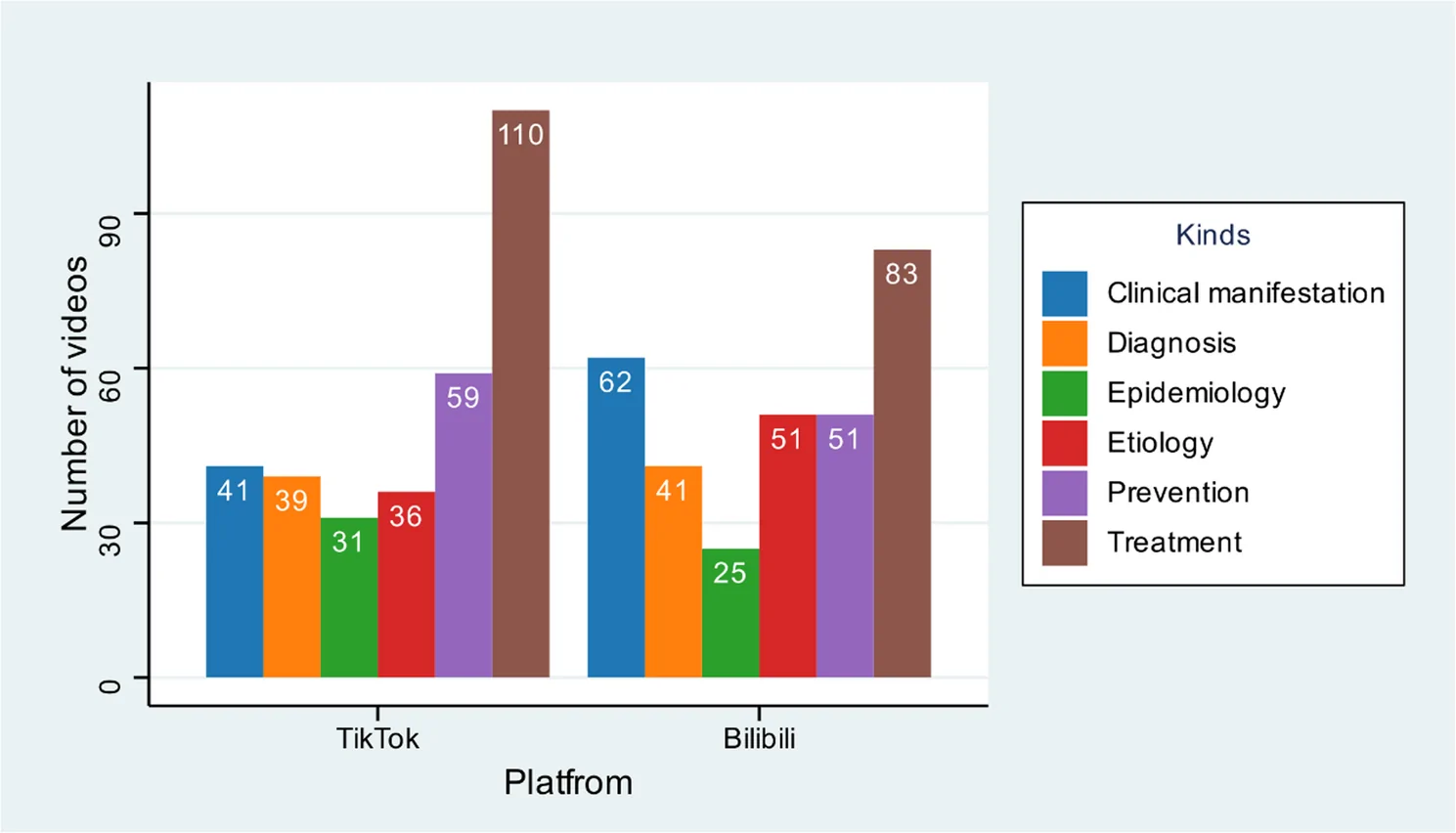
Distribution of video content categories on TikTok and Bilibili platforms

Thematic distribution analysis demonstrated that treatment-oriented content constituted the dominant focus on both platforms (Fig. 4). Information related to clinical manifestations and preventive guidance was comparatively less represented, while epidemiological and diagnostic content accounted for the smallest proportions. Overall, the content structure exhibited a pronounced imbalance characterized by an emphasis on therapeutic information and a relative underrepresentation of foundational medical knowledge.

### Quality and content differences by uploader type

**Fig. 5:**
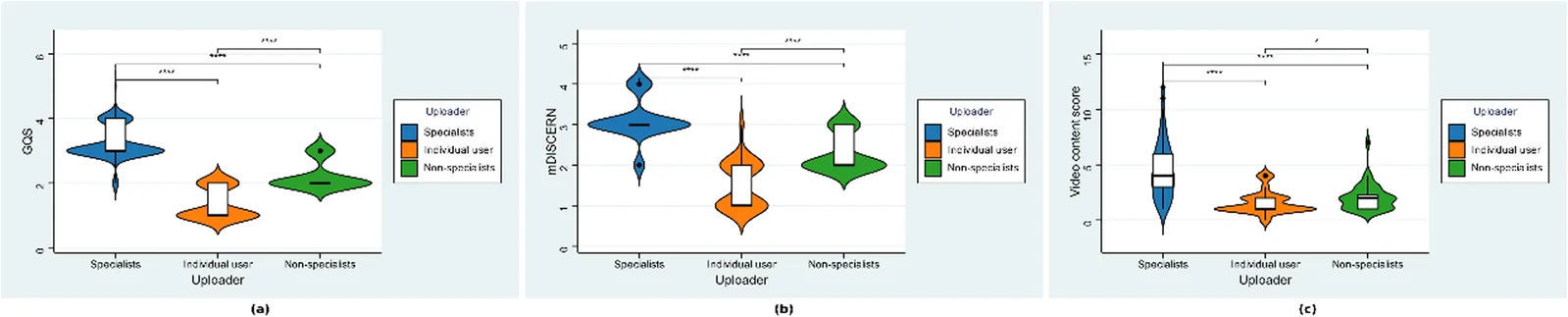
Uploader-type differences in quality (GQS), reliability (mDISCERN), and video content score. Notation: (**a**) Global Quality Scale (GQS) scores, (**b**) modified DISCERN (mDISCERN) scores, and (**c**) video content scores are compared among specialists, individual users, and non-specialists. Data are presented as violin plots with embedded boxplots. Statistical significance between groups is indicated (*P < 0.05, ****P < 0.0001)

Marked stratification effects were observed across uploader categories in terms of video quality, information reliability, and content completeness (Fig. 5, Table 5). Videos produced by specialist physicians and healthcare professionals achieved significantly higher Global Quality Scale (GQS) scores, modified DISCERN (mDISCERN) scores, and content completeness scores compared with those uploaded by non-specialist contributors and individual users. This consistent performance gradient reflects a stable professional advantage pattern and indicates a significant positive association between uploader expertise and the quality, credibility, and structural integrity of digital health information.

**Table 5:** Characteristics, quality, and reliability of prostatitis videos by different uploaders on TikTok and Bilibili

Variables	Individual user (*n* = 53)	Non-specialists (*n* = 36)	Specialists (*n* = 134)	*P*
Video length, M (Q₁, Q₃)	164.00 (112.00,313.00)	82.00 (62.50,132.50)	77.00 (48.00,162.00)	**< 0.001**
Likes, M (Q₁, Q₃)	27.00 (6.00,209.00)	40.00 (13.50,207.75)	113.00 (27.25,666.00)	**0.004**
Collections, M (Q₁, Q₃)	15.00 (4.00,143.00)	20.50 (11.75,86.75)	53.00 (9.25,327.75)	**0.023**
Comments, M (Q₁, Q₃)	5.00 (2.00,43.00)	4.50 (1.00,25.25)	15.00 (3.00,79.75)	0.126
Shares, M (Q₁, Q₃)	3.00 (0.00,32.00)	3.00 (0.00,26.25)	17.00 (3.25,90.25)	**0.001**
Video content score, M (Q₁, Q₃)	1.00 (1.00,2.00)	2.00 (1.00,2.25)	4.00 (3.00,6.00)	**< 0.001**
GQS, M (Q₁, Q₃)	1.00 (1.00,2.00)	2.00 (2.00,2.00)	3.00 (3.00,4.00)	**< 0.001**
mDISCERN, M (Q₁, Q₃)	1.00 (1.00,2.00)	2.00 (2.00,3.00)	3.00 (3.00,3.00)	**< 0.001**

*M* Median, *Q₁* 1st Quartile, *Q₃* 3rd Quartile

### Correlation analysis of engagement and quality

**Fig. 6:**
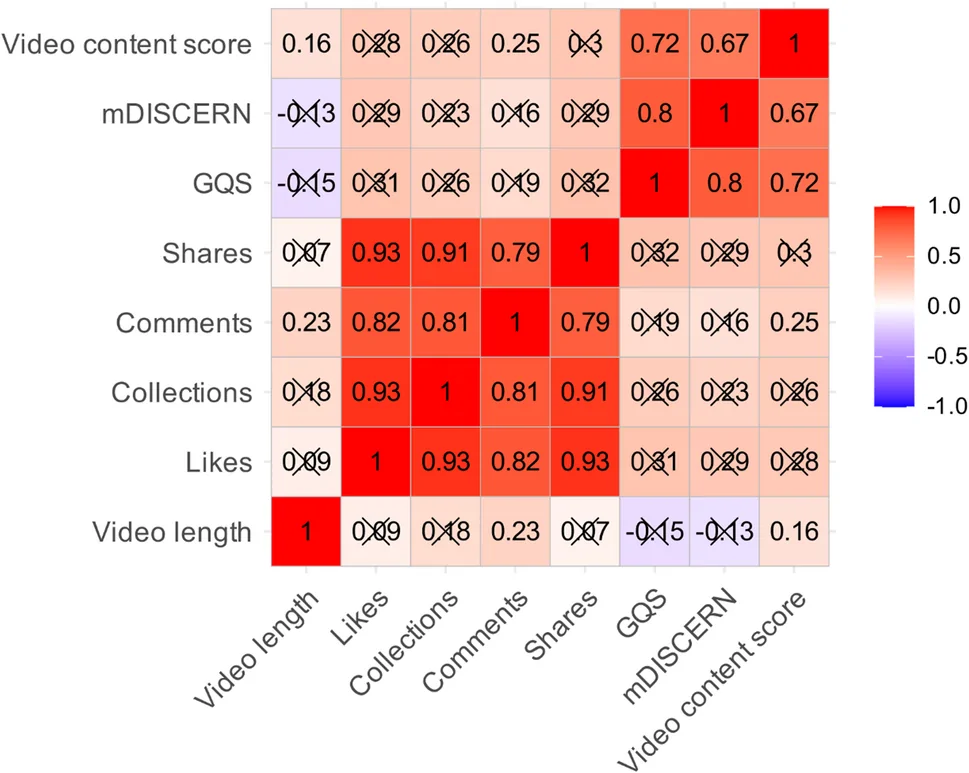
Correlation matrix of video engagement metrics and quality scores on TikTok and Bilibili. Notation: Heatmap showing Spearman correlation coefficients among video length, engagement metrics (likes, collections, comments, shares), Global Quality Scale (GQS), modified DISCERN (mDISCERN), and video content score. Color intensity reflects correlation strength and direction. Values marked with × indicate non-significant correlations (*P* > 0.05)

Correlation profiling revealed strong interrelationships among engagement metrics (likes, favorites, comments, and shares), with coefficients ranging from approximately 0.79 to 0.93, indicating high internal consistency of dissemination indicators (Fig. 6). A robust correlation was observed between GQS and mDISCERN scores (*r* = 0.80), while content completeness exhibited moderate-to-strong associations with both quality and reliability measures (GQS: *r* = 0.72; mDISCERN: *r* = 0.67). In contrast, engagement metrics and video duration demonstrated only weak or negligible relationships with quality-related indicators, suggesting that dissemination popularity is not a reliable proxy for informational rigor or scientific credibility.

## Discussion

Prostatitis, particularly chronic prostatitis/chronic pelvic pain syndrome (CP/CPPS), remains a common yet challenging condition in urological practice, largely due to its complex etiology, heterogeneous clinical presentation, and the absence of a single definitive treatment strategy. In recent years, short-video platforms have become an increasingly important source of health information for patients with prostatitis. Through a systematic analysis of prostatitis-related videos on TikTok and Bilibili, the present study provides insight into the quality, structure, and clinical relevance of online information encountered by patients prior to or alongside medical consultation.

Unlike many previous studies that primarily reported overall quality scores of online health information, the present study attempted to interpret prostatitis-related short-video content from a urological clinical perspective by integrating information quality, reliability, uploader characteristics, engagement patterns, and disease-specific content structure. This approach is particularly relevant for prostatitis and chronic prostatitis/chronic pelvic pain syndrome because patient misunderstanding is not limited to whether information is generally “high quality” or “low quality,” but also depends on whether the content provides an appropriate diagnostic framework, explains the chronic and multifactorial nature of the disease, and avoids reinforcing unrealistic expectations of rapid or definitive cure. Therefore, the clinical value of this study lies in identifying not only the general quality limitations of short-video content but also the specific structural gaps that may influence patient expectations, clinical communication, and long-term adherence to multimodal management.

With respect to engagement dynamics, this study demonstrates a generally high level of user interaction, with strong correlations among likes, favorites, comments, and shares, reflecting the intrinsic coherence of platform dissemination mechanisms. Platform-based comparisons further revealed that TikTok exhibits significantly higher diffusion-oriented engagement indicators, particularly in likes and shares, whereas no significant differences were observed for favorites and comments, indicating structural differences in dissemination logic and user participation patterns between platforms. Previous research has shown that TikTok prioritizes algorithm-driven recommendation systems and high-frequency interaction feedback loops, facilitating high-exposure and high-engagement dissemination cycles, whereas Bilibili functions more as a knowledge-oriented community ecosystem in which user behavior emphasizes cognitive engagement, comprehension, and information accumulation rather than rapid diffusion and visibility expansion [18–20]. Importantly, the present findings further indicate that, within the digital dissemination context of chronic urological diseases such as prostatitis, high engagement does not necessarily translate into high-quality information acquisition. From a urological clinical perspective, this mismatch suggests that patients may be more likely to encounter highly visible but medically limited information, which can influence treatment expectations and complicate physician–patient communication. These observations underscore the importance of promoting evidence-based, professionally generated content in digital environments to support accurate patient understanding and informed decision-making [21].

At the level of content structure, this study demonstrates a pronounced concentration of video themes on treatment-related information, accompanied by a substantial underrepresentation of epidemiological knowledge, diagnostic content, and disease cognition, reflecting a structural imbalance characterized by “treatment dominance and cognitive insufficiency.” From a urological perspective, such imbalance carries important clinical risk implications, particularly for chronic prostatitis/chronic pelvic pain syndrome (CP/CPPS), in which effective management does not rely on single pharmacological interventions but rather on accurate phenotyping, differential diagnosis, and long-term comprehensive management strategies, including pain modulation, pelvic floor rehabilitation, psychological interventions, and lifestyle modification [6–24]. In the absence of sustained disease cognition and diagnostic frameworks, patients may develop unrealistic expectations for rapid medication-based solutions or repeated healthcare seeking, which may complicate subsequent clinical communication regarding diagnostic evaluation, antibiotic expectations, and long-term multimodal management [25, 26]. Accordingly, health communication on short-video platforms should move beyond a treatment-centered dissemination model and instead strengthen the structured communication of disease classification, diagnostic pathways, symptom recognition, and long-term management principles, thereby facilitating the construction of comprehensive public disease cognition frameworks and promoting more rational health-seeking behaviors.

With regard to information quality and reliability, the overall moderate performance observed in this study indicates that health content on short-video platforms continues to be characterized by insufficient source attribution, incomplete evidence presentation, and limited structural organization. Similar patterns have been consistently reported across multiple disease domains, where short-form video content prioritizes visual appeal and dissemination efficiency over scientific rigor and evidence-based structuring [27–29]. In platform-level comparisons, TikTok videos achieved higher GQS and mDISCERN scores than those on Bilibili, which may partly reflect a higher proportion of professionally affiliated accounts and more standardized content production. However, the inherently condensed format of short videos may limit the depth with which complex urological conditions can be accurately explained. From a urological clinical perspective, such limitations are particularly relevant for prostatitis, where diagnostic reasoning and long-term management principles are essential for appropriate patient understanding. These findings highlight the need to encourage more structured, evidence-based communication approaches in digital settings to support accurate disease perception and informed clinical decision-making.

Across uploader categories, videos produced by specialist physicians and healthcare professionals demonstrated significantly superior performance in terms of video quality, information reliability, and content completeness compared with those uploaded by non-specialist contributors and individual users, reflecting a stable and consistent professional advantage effect. This finding is highly concordant with existing literature, which identifies medical background as a core determinant of health information credibility [30–33]. Notably, however, even content originating from professional sources remained at a moderate overall quality level, indicating that short-video platforms have not yet established a highly standardized system for high-quality health content production. For conditions such as prostatitis, which are characterized by complex etiologies, widespread public misconceptions, and elevated patient anxiety, experience-based health communication alone is insufficient to meet the demands of effective health education. Accordingly, platforms should institutionally strengthen professional content certification, provide structural support for organizational and institutional accounts, and implement systematic content quality review mechanisms to enhance the standardization and evidence-based integrity of professional content, while simultaneously promoting public health literacy and rational information appraisal capacities, thereby preventing the uncritical equating of professional identity labels with content quality assurance.

Correlation analyses further exposed structural deficiencies at the level of dissemination mechanisms. Engagement indicators were strongly intercorrelated, whereas their associations with video quality, information reliability, and content completeness were weak; similarly, video duration failed to demonstrate a stable positive relationship with quality and reliability measures, consistent with findings from previous studies on meningitis- and systemic lupus erythematosus–related video content [34, 35]. These patterns indicate that dissemination popularity does not reliably reflect the scientific value of health information and that highly engaged content does not necessarily correspond to high-quality health communication. This phenomenon aligns with broader social media research demonstrating that highly transmissible content tends to spread more easily but is not inherently more accurate, suggesting that algorithmic recommendation systems may systematically amplify the visibility of low-quality information [36, 37]. From a urological clinical perspective, this suggests that patients may be exposed to high-popularity yet low-quality information, which could shape expectations before consultation and complicate subsequent shared decision-making. Accordingly, future platform algorithm design should incorporate quality-oriented dimensions such as structural clarity, evidence attribution, and professional credibility into recommendation logic, thereby reconstructing health information dissemination pathways toward scientifically grounded and clinically meaningful communication.

Overall, this study demonstrates that prostatitis-related information on short-video platforms is characterized by moderate quality, a strong treatment-oriented focus, and insufficient coverage of disease cognition and diagnostic principles. Although professional sources provide relatively higher-quality content, a clear disconnect remains between information popularity and clinical value. These findings suggest that short-video platforms influence how patients understand prostatitis before clinical consultation, highlighting the need for more structured, evidence-based digital patient education to support appropriate expectations and long-term management in urological practice.

This study has several limitations that should be acknowledged. First, the analysis was limited to TikTok and Bilibili and did not include other short-video or video-sharing platforms such as YouTube or Instagram, which may limit the generalizability of the findings across different cultural and healthcare contexts. In addition, both platforms were accessed in the Chinese domestic short-video environment, and Bilibili is predominantly used in China; therefore, the findings may primarily reflect platform-specific and region-specific patterns of prostatitis-related health communication rather than fully representing global social media perspectives. Second, videos were collected at a single time point, and the ranking and visibility of short-video content may change dynamically over time due to platform algorithms and user engagement. Third, although the general keyword “prostatitis” was selected to capture the broader information environment, videos using more specific terms such as “chronic prostatitis” or “CP/CPPS” may not have been fully retrieved. Fourth, although standardized assessment tools, inter-rater reliability analysis, and a dual-reviewer approach were employed, the evaluation of video quality and content completeness inevitably involved a degree of subjectivity. Finally, this study evaluated video content only and did not directly assess patient behavior, prescribing patterns, healthcare-seeking decisions, or clinical outcomes; therefore, the clinical implications should be interpreted as potential influences on patient expectations and clinical communication rather than as direct causal effects. Future studies should incorporate larger, multi-platform datasets, repeated sampling at different time points, multi-keyword search strategies, longitudinal designs, and patient-centered outcomes to better clarify the clinical implications of online information exposure in urological practice.

## Conclusion

This study demonstrates that prostatitis-related content on short-video platforms is widely accessible but often limited in clinical depth and structural completeness. Although videos produced by healthcare professionals show relatively better quality and reliability, substantial gaps remain, particularly in the communication of disease cognition, diagnostic principles, and long-term management strategies. Given the complex nature of prostatitis and CP/CPPS, such informational shortcomings may influence patient expectations and may complicate subsequent clinical communication and shared decision-making in clinical practice.These findings highlight the need for urologists to play a more active role in digital patient education and to address misinformation encountered by patients outside the clinical setting, thereby improving shared decision-making and long-term disease management.

## Supplementary Information

Supplementary Material 1.

## Data Availability

The data analyzed in this study are derived from publicly available videos on TikTok and Bilibili. The datasets generated during the current study are available from the corresponding author upon reasonable request.
