# Supplementary material for: Quality, content structure, and reliability of prostatitis-related information on short-video platforms: a cross-platform analysis of TikTok and Bilibili
Source: BMC Urol. 2026 Jun 17;26:211. doi: 10.1186/s12894-026-02225-y (PMC13520534; doi:10.1186/s12894-026-02225-y)
Supplement: Supplementary file 1 [file 12894_2026_2225_MOESM1_ESM.docx]

| **Supplementary Table S1. Inter-rater reliability for video evaluation** | | |
| --- | --- | --- |
| Assessment item | Reliability metric | Value |
| GQS | ICC | 0.963 |
| mDISCERN | ICC | 0.93 |
| Video content total score | ICC | 0.982 |
| Epidemiology | Weighted Cohen’s κ | 0.937 |
| Etiology | Weighted Cohen’s κ | 0.951 |
| Clinical manifestation | Weighted Cohen’s κ | 0.899 |
| Diagnosis | Weighted Cohen’s κ | 0.972 |
| Treatment | Weighted Cohen’s κ | 0.983 |
| Prevention | Weighted Cohen’s κ | 0.994 |

**Abbreviations:** GQS, Global Quality Scale; mDISCERN, modified DISCERN; ICC, intraclass correlation coefficient; κ, kappa.
**Note:** ICCs were calculated for GQS, mDISCERN, and total video content score. Weighted Cohen’s κ coefficients were calculated for the six ordinal content-domain scores.
